# Adherence to growth hormone (GH) therapy in naïve to treatment GH-deficient children: data of the Italian Cohort from the Easypod Connect Observational Study (ECOS)

**DOI:** 10.1007/s40618-019-01046-1

**Published:** 2019-04-09

**Authors:** C. Centonze, C. Guzzetti, G. Orlando, S. Loche, the Italian ECOS Investigators, C. Angeletti, F. Antoniazzi, S. Bernasconi, G. M. Cardinale, M. Caruso-Nicoletti, L. Cavallo, S. Cianfarani, G. Citro, F. De Luca, S. Della Casa, M. Di Pietro, P. Garofalo, C. Giordano, N. A. Greggio, M. R. Licenziati, M. Maghnie, M. Parpagnoli, L. Persani, S. Pesce, M. Sacco, M. Salerno, L. Tafi

**Affiliations:** 1 Medical Affairs Department, Merck Serono S.p.A., Rome, Italy; 2 SSD di Endocrinologia Pediatrica e, Centro Screening Neonatale, Ospedale Pediatrico Miccrocitemico “A. Cao”, AO Brotzu, Via Jenner, 09121 Cagliari, Italy

**Keywords:** ECOS, Short stature, Children, Growth hormone, GHD

## Abstract

**Background:**

With the use of non-objective measurement, adherence to growth hormone (GH) therapy has been reported suboptimal in a large proportion of patients, and poor adherence has been shown to affect short-term growth response in patients receiving GH treatment.

**Objective:**

The Easypod™ electronic device allows objective measurement of adherence. In this study, we report 3-year prospective adherence data of the Italian cohort of naïve GH deficient (GHD) children extrapolated from the Easypod Connect Observational Study (ECOS) database.

**Patients and methods:**

Seventy-three GHD children naïve to GH treatment were included in the analysis. 22 Italian centers participated in the study.

**Results:**

Mean adherence rate was consistently above 85% across the 3-year observation period. Particularly, mean adherence was 88.5%, 86.6%, and 85.7% after 1, 2 and 3 years, respectively. Mean (± SD) height-SDS increase after the first year was 0.41 (± 0.38).

**Conclusions:**

The majority of naïve GHD children starting GH treatment with Easypod maintained an adherence rate > 85% up to 3 years. Easypod is a useful tool to follow-up patients’ adherence allowing timely intervention to improve optimal treatment for these patients.

## Introduction

Human recombinant growth hormone (h-rGH) is currently used to treat a variety of clinical conditions associated with short stature, including GH deficiency (GHD), Turner syndrome, Prader–Willi syndrome, children born small for gestational age (SGA), and children with chronic renal failure [1]. The growth response to GH therapy is influenced by a number of factors which include, among others, the initial diagnosis, the age of the patient, the dose of GH administered as well as individual responsiveness [2–4]. Although adherence to medication is a well-known clinical problem affecting several chronic diseases, measuring adherence is generally difficult and mostly based on patient self-reported questionnaires, prescription records, or vials counting [5]. With the use of non-objective measurement, adherence to GH therapy has been reported suboptimal in up to 77% of patients in different studies [6–12]. Poor adherence has been shown to be related to several factors including lower socio-economic or educational status, injections considered difficult, lack of choice of injection device, use of conventional syringe rather than automatic pen injection device, and discomfort with injections [13]. Furthermore, poor adherence has been shown to affect short-term growth response in patients receiving GH treatment [11, 12, 14].

The easypod™ electronic device allows objective measurement of adherence, and short-term studies have recently shown a good acceptance and adherence in patients taking h-rGH via this device [15–18]. Easypod allows transmission of injections’ data (real time recorded dose and timing) by a wireless transmitter to physician, thus allows to examine individual patients’ adherence remotely in real time settings. Easypod Connect Platform (ECP), as a whole, provides an e-health solution to improve patient management by remote examination and control of adherence to h-r.

The Easypod Connect Observational Study (ECOS) is a prospective international 5-year investigation designed to assess adherence to GH treatment in patients taking h-rGH via the Easypod device for several conditions. Data from this large international study including 1203 patients from 24 different countries have been recently published, and shown that median adherence rate in the entire cohort was maintained over 80% in the first 3 years, and declined thereafter [19]. This study has also reported adherence rate across the 5-year observation period in the subgroup of naïve GHD patients of different ethnic origin, and shown that mean adherence rate in the first 2 years was > 80%, and > 75% in the third year irrespective of the cause of GHD. In this study, we report 3-year prospective adherence data of the Italian cohort of naïve GHD children extrapolated from the ECOS database.

## Patients and methods

Seventy-three GHD children naïve to GH treatment were recruited in 22 Italian centers and included in the analysis. Their main clinical characteristics are summarized in Table 1. The diagnosis of GHD was performed in each participating center according to standard endocrine practice. Seventy children had idiopathic GHD, two had organic GHD and one had congenital GHD. The study was approved by the local ethical committees, and written informed consent was obtained from the patients and/or from their legal guardians prior to their enrollment in the study. As reported in the large ECOS study [19], patients were enrolled in the database and after an initial baseline visit, they were seen 1–4 times/year. Adherence rate was derived from the Easypod device and calculated as the percentage of injections recorded versus prescribed.

**Table 1:** Main clinical characteristics of the children studied

Age, years	
Mean (SD)	9.78 (3.20)
Median	10.00
Q1; Q3	8.00; 12.00
Min; max	1.0; 15.0
Sex, *n* (%)	
Female	35 (47.9)
Male	38 (52.1)
Change in height SDS (*n* = 70)	
Mean (SD)	0.42 (0.38)
Median	0.41
Q1; Q3	0.18; 0.61

## Results

**Fig. 1:**
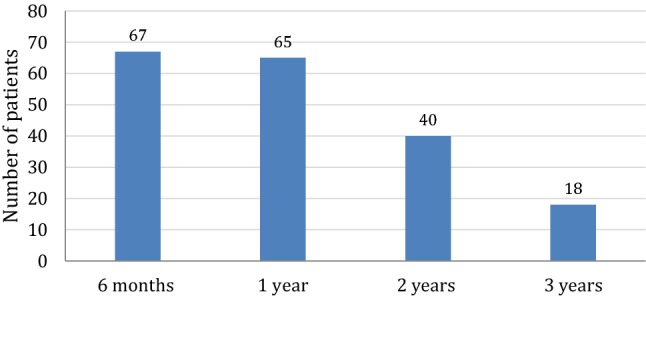
Number of patients with prospective adherence data over the study period

**Fig. 2:**
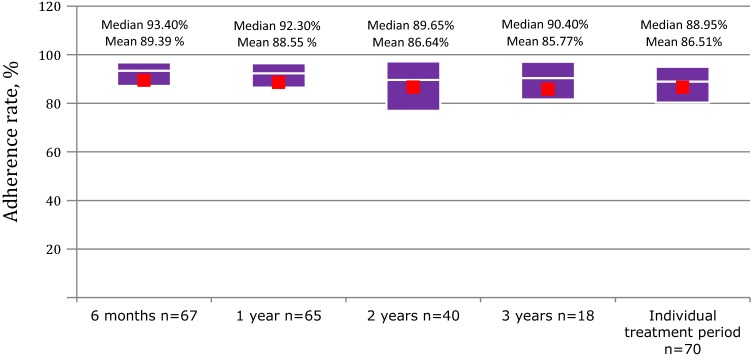
Treatment adherence rates over time in the Easypod™ adherence data analysis. Boxes show Q1 and Q3, with median as white line, and mean as red squares

Adherence data were available for 65 patients after 1 year, for 40 patients after 2 years, and for 18 patients after 3 years (Fig. 1). Mean adherence rate was consistently above 85% across the 3-year observation period (Fig. 2). Particularly, mean adherence was 88.5%, 86.6%, and 85.7% after 1, 2 and 3 years, respectively. Mean adherence rate for individual treatment period (from the beginning of treatment to the last complete week of available data for each patient) was 86.51% (Fig. 2). Mean (± SD) height-SDS increase after the first year was 0.41 (± 0.38) (Table 1). We found no significant correlation between the level of adherence and growth outcome after the first year of treatment.

## Discussion

We have shown in this study that in the ECOS Italian cohort of GHD patients naïve to GH therapy the mean level of adherence is maintained > 85% for up to 3 years. The analysis of this subset of patients from a single country confirms the observation of the global ECOS data [19]. The recent large ECOS study has shown, in fact, that in naïve GHD patients, the adherence rate is maintained > 80% in the first 2 years, and declines thereafter. Of note, in the subset of patients of the italian cohort of this study, mean adherence rate was slightly better, and did not decline in the third year as in the large ECOS study, confirming our previous observation that children with GHD treated with r-hGH via the Easypod device generally have a good adherence to treatment [17]. In our previous short-term study, we recorded high levels of adherence in 97 patients taking r-hGH, with 57% of them having an adherence rate > 92%. Other short-term studies have confirmed good adherence in pediatric patients receiving h-rGH via the Easypod device, ranging from 87.5 to 99% [16–18, 20].

In the present study, treatment was associated with the expected increase in height. The observed variability of the first year response is in line with previous findings obtained from analyses of large databases [21, 22]. The variability of the growth response is a well known phenomenon, possibly related to the heterogeneity of patients in term of severity of GHD as well as individual responsiveness [3].

The diagnosis of growth hormone deficiency (GHD) is currently based on clinical and auxological assessment, with the supporting evidence from biochemical and MRI studies [23]. The diagnostic workup in a patient with suspected GHD includes evaluation of GH secretion by means of stimulation testing. The Italian Medicine Agency (AIFA) regulates the modalities of growth hormone prescription to GHD children, as well as the criteria for diagnosis [24]. Complying with the prescription criteria is mandatory for endocrinologists who can only prescribe GH within hospitals specifically accredited by government. Therefore, all GHD patients included in the ECOS in Italy fulfill the same national standards for diagnostic criteria. Furthermore, all patients of our study are of Caucasian origin. Based on these premises, our sample population can be considered homogeneous since our patients share the same endocrine phenotype as well as ethnic origin. In this regard, it should be pointed out that poor adherence has been correlated with ethnic origin [12] as well as with training by non-hospital staff or no training rather than training by hospital staff [6].

In short-term studies poor adherence has been shown to be related with the growth outcome [11, 12, 14]. The global ECOS study [19] has reported a positive correlation between adherence rate and growth response in the GHD patients. We found no correlation between the level of adherence and the growth outcome after the first year, possibly due to the small number of subjects and to the fact that adherence values were skewed toward high positive levels. Our results are in agreement with those recently reported by Van Dommelen et al. [25] who showed that average adherence in the first year was not associated with first year growth response in children with GHD but was highly correlated with growth response in the second year and 0–2 years in total.

In conclusion, we have shown that the majority of naïve GHD children starting GH treatment with Easypod in Italy maintained an adherence rate > 85% up to 3 years. Easypod is a useful tool to follow-up patients’ adherence allowing timely intervention to improve optimal treatment for these patients.
